# Progress Toward Hepatitis B Control and Elimination of Mother-to-Child Transmission of Hepatitis B Virus — World Health Organization African Region, 2016–2021

**DOI:** 10.15585/mmwr.mm7229a2

**Published:** 2023-07-21

**Authors:** Hyacinte J. Kabore, Xi Li, Mary M. Alleman, Casimir M. Manzengo, Mutale Mumba, Joseph Biey, Gilson Paluku, Ado M. Bwaka, Benido Impouma, Rania A. Tohme

**Affiliations:** ^1^Vaccine-Preventable Disease Unit, World Health Organization Regional Office for Africa, Brazzaville, Republic of the Congo; ^2^Global Immunization Division, Global Health Center, CDC; ^3^HIV, Tuberculosis, Hepatitis Unit, Inter-country Support Team, World Health Organization, Libreville, Gabon; ^4^Vaccine Preventable Diseases Unit, World Health Organization Inter-country Support Team - East and South, Harare, Zimbabwe; ^5^Vaccine Preventable Diseases Unit, World Health Organization Inter-country Support Team - West, Ouagadougou, Burkina Faso; ^6^Vaccine Preventable Diseases Unit, World Health Organization Inter-country Support Team - Central, Libreville, Gabon; ^7^Universal Health Coverage/Communicable & Non-Communicable Diseases, World Health Organization Regional Office for Africa, Brazzaville, Republic of the Congo.

**Summary**

**What is already known about this topic?**

In 2019, the World Health Organization African Region (AFR) accounted for 66% of all new chronic hepatitis B virus (HBV) infections. Chronic HBV infection is the leading causes of cirrhosis and liver cancer.

**What is added by this report?**

By 2021, all 47 AFR countries provided 3 doses of hepatitis B vaccine (HepB3) to infants, and 14 (30%) provided a birth dose (HepB-BD). By December 2021, 16 (34%) countries achieved ≥90% HepB3 coverage; two (4%) achieved ≥90% timely HepB-BD coverage. Four countries achieved hepatitis B control; none achieved elimination of mother-to-child transmission (EMTCT).

**What are the implications for public health practice?**

Introduction of HepB-BD, improving HepB3 and HepB-BD coverage, and monitoring implementation of EMTCT interventions are essential to accelerating progress toward hepatitis B control and EMTCT in AFR.

## Abstract

Chronic hepatitis B virus (HBV) infection is one of the leading causes of cirrhosis and liver cancer. In 2019, approximately 1.5 million persons newly acquired chronic HBV infection; among these, 990,000 (66%) were in the World Health Organization (WHO) African Region (AFR). Most chronic HBV infections are acquired through mother-to-child transmission (MTCT) or during early childhood, and approximately two thirds of these infections occur in AFR. In 2016, the World Health Assembly endorsed the goal of elimination of mother-to-child transmission (EMTCT) of HBV, documented by ≥90% coverage with both a timely hepatitis B vaccine (HepB) birth dose (HepB-BD) and 3 infant doses of HepB (HepB3), and ≤0.1% hepatitis B surface antigen (HBsAg) seroprevalence among children aged ≤5 years. In 2016, the WHO African Regional Committee endorsed targets for a 30% reduction in incidence (≤2% HBsAg seroprevalence in children aged ≤5 years) and ≥90% HepB3 coverage by 2020. By 2021, all 47 countries in the region provided HepB3 to infants beginning at age 6 weeks, and 14 countries (30%) provided HepB-BD. By December 2021, 16 (34%) countries achieved ≥90% HepB3 coverage, and only two (4%) achieved ≥90% timely HepB-BD coverage. Eight countries (17%) conducted nationwide serosurveys among children born after the introduction of HepB to assess HBsAg seroprevalence: six countries had achieved ≤2% seroprevalence, but none had achieved ≤0.1% seroprevalence among children. The development of immunization recovery plans following the COVID-19 pandemic provides an opportunity to accelerate progress toward hepatitis B control and EMTCT, including introducing HepB-BD and increasing coverage with timely HepB-BD and HepB3 vaccination. Representative HBsAg serosurveys among children and a regional verification body for EMTCT of HBV will be needed to monitor progress.

## Introduction

In 2019, approximately 1.5 million persons newly acquired chronic hepatitis B virus (HBV) infection; among these, 990,000 (66%) were in the World Health Organization (WHO) African Region (AFR)* (*1*). Because most chronic HBV infections are acquired through mother-to-child transmission (MTCT) or during early childhood (*2*), WHO recommends that all newborns receive a dose of hepatitis B vaccine (HepB) within 24 hours of birth (hepatitis B vaccine birth dose [HepB-BD]) followed by 2 or 3 doses^†^ of HepB during the first year of life (*2*). In 2016, the World Health Assembly endorsed the goal of eliminating viral hepatitis as a public health threat by 2030, including the elimination of mother-to-child transmission (EMTCT) of HBV, documented by demonstration of ≥90% coverage with both a timely^§^ HepB-BD and 3 doses of HepB (HepB3), and ≤0.1% hepatitis B surface antigen (HBsAg)^¶^ seroprevalence among children aged ≤5 years (*3*). In 2016, the WHO African Regional Committee endorsed two targets for hepatitis B control: 1) 30% reduction in incidence (equating to HBsAg prevalence of ≤2% in children aged ≤5 years), and 2) ≥90% HepB3 coverage by 2020. In 2021, AFR countries endorsed a call to develop strategies for elimination of MTCT of HBV, including increasing HepB-BD and HepB3 coverage and improving access to antenatal care and quality delivery services (*4*,*5*). This report describes progress made during 2016–2021 to achieve hepatitis B control and elimination of MTCT of HBV in AFR.

## Methods

Information on country immunization activities was obtained by review of administrative** or official^††^ HepB coverage data reported to WHO and UNICEF that generate annual country vaccination coverage estimates. To identify HBsAg seroprevalence surveys conducted in AFR, a MEDLINE literature review was conducted using the following search criteria (Afro country names), and (“hepatitis B” OR “HBV”) AND (2016/10/01:3000/12/31[Date - Publication]) AND (survey OR serosurvey OR serosurveillance OR seroepidemiology OR prevalence OR seroprevalence). Population-based surveys including the Population based HIV Impact Assessment (PHIA) surveys and Demographic Health Survey (DHS) were also used. This activity was reviewed by CDC and was conducted with applicable federal laws and CDC policy.^§§^

## Results

### Immunization Activities

By 2014, all 47 countries in AFR had introduced HepB3 infant vaccination (Table 1). By December 2021, 14 (30%) countries provided HepB-BD, eight (57%) of which were in the West subregion.^¶¶^ Although 10 countries had introduced HepB-BD before 2016, only four (Benin, Côte d’Ivoire, Equatorial Guinea, and Senegal) introduced HepB-BD during 2016–2021. During this period, regional HepB3 coverage ranged from 75% in 2019 to 71% in 2021. Eighteen (38%) countries reached ≥90% HepB3 coverage in 2016; this number peaked at 20 (43%) in 2018; by 2021, the number of countries with ≥90% HepB3 coverage had declined to 16 (34%); nine of these countries were in the East and South subregions. Regional HepB-BD coverage increased from 10% in 2016 to 17% in 2021. During 2016–2021, Algeria and Cabo Verde reached HepB-BD coverage of ≥90%, and Namibia and Senegal achieved ≥50% coverage.

**TABLE 1:** Year of hepatitis B vaccine introduction, hepatitis B vaccination schedule and estimated coverage* with the third vaccine dose, a timely administered hepatitis B vaccine birth dose,^†^ and rates of institutional delivery, by country — World Health Organization African Region, 2016–2021

Region, country	Year of introduction		HepB Schedule	HepB3 coverage, %						Timely HepB-BD coverage, %						Rates of institutional delivery, % (most recent source and year)
	HepB	HepBD		2016	2017	2018	2019	2020	2021	2016	2017	2018	2019	2020	2021	
**Central subregion**																
Angola	2006	2015	B, 2, 4, 6 mos	55	52	59	53	47	41	NR	NR	NR	NR	NR	NR	45.6 (DHS 2015–2016)
Burundi	2004	—	6, 10, 14 wks	94	91	90	93	93	94	NA	NA	NA	NA	NA	NA	83.9 (DHS 2016–2017)
Cameroon	2005	—	6, 10, 14 wks	75	74	67	67	69	69	NA	NA	NA	NA	NA	NA	67.0 (DHS 2018)
Central African Republic	2003	—	6, 10, 14 wks	42	42	42	42	42	42	NA	NA	NA	NA	NA	NA	58.3 (MICS 2018–2019)
Chad	2003	—	6, 10, 14 wks	41	41	46	50	52	58	NA	NA	NA	NA	NA	NA	27.2 (MICS 2019)
Democratic Republic of the Congo	2003	—	6, 10, 14 wks	70	71	71	73	70	65	NA	NA	NA	NA	NA	NA	81.5 (MICS 2017–2018)
Equatorial Guinea	2003	2018	B, 6, 10, 14 wks, 18 mos	53	53	53	53	53	53	NA	NA	NA	NR	NR	NR	67.3 (DHS 2011)
Gabon	2003	—	6, 10, 14 wks	75	75	70	70	63	75	NA	NA	NA	NA	NA	NA	90.2 (DHS 2012)
Republic of the Congo	2003	—	8, 12, 16 wks	71	69	75	79	73	77	NA	NA	NA	NA	NA	NA	91.5 (MICS 2014–2015)
Sao Tome and Principe	2003	2010^§^	B, 6, 10, 14 wks	96	95	95	95	96	97	NA	NA	NA	95	82	69	95.4 (MICS 2019)
**East and South subregion**																
Botswana	1994	1998	B, 2, 3, 4 mos	95	95	95	95	95	95	NR	NR	NR	NR	NR	NR	99.7 (Other NS 2015)
Comoros	2003	—	6, 10, 14 wks	91	91	91	91	87	85	NA	NA	NA	NA	NA	NA	76.1 (DHS–MICS 2012)
Eritrea	2002	—	6, 10, 14 wks	95	95	95	95	95	95	NA	NA	NA	NA	NA	NA	33.7 (Other NS 2010)
Eswatini	1996	—	6, 10, 14 wks	90	90	90	90	83	77	NA	NA	NA	NA	NA	NA	87.7 (MICS 2014)
Ethiopia	2007	—	6, 10, 14 wks	66	68	68	68	71	65	NA	NA	NA	NA	NA	NA	47.5 (DHS (Mini) 2019)
Kenya	2001	—	6, 10, 14 wks	89	82	92	91	91	91	NA	NA	NA	NA	NA	NA	61.2 (DHS 2014)
Lesotho	2003	—	6, 10, 14 wks	87	87	87	87	87	87	NA	NA	NA	NA	NA	NA	89.4 (MICS 2018)
Madagascar	2002	—	6, 10, 14 wks	68	65	65	68	66	55	NA	NA	NA	NA	NA	NA	38.7 (MICS 2018)
Malawi	2002	—	6, 10, 14 wks	84	88	92	95	90	93	NA	NA	NA	NA	NA	NA	96.7 (MICS 2019–2020)
Mauritius	1996	1996^§^	R^¶^, 6, 10, 14 wks, 18 mos	72	96	97	97	93	92	NA	NA	NA	NA	NA	NA	98.4 (MoH 2003)
Mozambique	2001	—	6, 10, 14 wks	88	88	88	88	79	61	NA	NA	NA	NA	NA	NA	54.8 (DHS 2011)
Namibia	2009	2014	B, 6, 10, 14 wks	85	88	89	87	93	93	85	81	76	81	86	86	87.4 (DHS 2013)
Rwanda	2002	—	6, 10, 14 wks	98	98	97	98	91	88	NA	NA	NA	NA	NA	NA	93.1 (DHS 2019–2020)
Seychelles	1996	—	3, 4, 5 mos	96	97	99	99	97	94	NA	NA	NA	NA	NA	NA	NR
South Africa	1995	—	6, 10, 14 wks, 18 mos	85	84	82	85	84	86	NA	NA	NA	NA	NA	NA	95.9 (DHS 2016)
South Sudan	2014	—	6, 10, 14 wks	45	47	49	49	49	49	NA	NA	NA	NA	NA	NA	11.5 (SHHS 2010)
Tanzania	2002	—	6, 10, 14 wks	92	90	89	89	86	81	NA	NA	NA	NA	NA	NA	62.6 (DHS 2015–2016)
Uganda	2002	—	6, 10, 14 wks	93	94	93	93	89	91	NA	NA	NA	NA	NA	NA	73.4 (DHS 2016)
Zambia	2005		6, 10, 14 wks	95	94	90	88	84	91	NA	NA	NA	NA	NA	NA	83.8 (DHS 2018–2019)
Zimbabwe	1994	—	6, 10, 14 wks	90	89	89	90	86	86	NA	NA	NA	NA	NA	NA	85.5 (MICS 2019)
**West subregion**																
Algeria	2001	2001	B, 2, 4, 12 mos	91	91	91	91	91	91	99	99	99	99	99	99	98.6 (MICS 2018–2019)
Benin	2002	2020	B, 6, 10, 14 wks	76	76	76	76	72	76	NA	NA	NA	NA	21	71	83.9 (DHS 2017–2018)
Burkina Faso	2006	—	8, 12, 16 wks	91	91	91	91	91	91	NA	NA	NA	NA	NA	NA	82.2 (Other NS 2015)
Cabo Verde	2002	2002	B, 2, 4, 6, 18 mos	96	97	99	97	94	94	96	96	97	96	96	96	97.0 (IDSR 2018)**
Côte d'Ivoire	2003	2019	B, 6, 10, 14 wks	87	83	84	81	75	76	NA	NA	NA	9	62	66	69.8 (MICS 2016)
The Gambia	1995	1999	B, 2, 3, 4 mos	95	92	93	88	86	82	NR	NR	NR	NR	NR	25	83.7 (DHS 2019–2020)
Ghana	2002	—	6, 10, 14 wks	93	99	97	97	94	98	NA	NA	NA	NA	NA	NA	77.9 (MICS 2017–2018)
Guinea	2006	—	6, 10, 14 wks	47	45	47	47	47	47	NA	NA	NA	NA	NA	NA	52.6 (DHS 2018)
Guinea-Bissau	2008	—	6, 10, 14 wks	85	79	82	78	74	67	NA	NA	NA	NA	NA	NA	50.4 (MICS 2018–2019)
Liberia	2008	—	6, 10, 14 wks	73	80	80	70	65	66	NA	NA	NA	NA	NA	NA	79.8 (DHS 2019–2020)
Mali	2002	—	6, 10, 14 wks	76	77	77	77	70	77	NA	NA	NA	NA	NA	NA	66.8 (DHS 2018)
Mauritania	2005	2013	B, 6, 10, 14 wks	74	76	77	80	72	68	NR	NR	NR	NR	NR	NR	69.3 (MICS 2015)
Niger	2008	—	6, 10, 14 wks	80	85	79	81	81	82	NA	NA	NA	NA	NA	NA	44.3 (ENAFEME 2021)**
Nigeria	2004	2004	B, 6, 10, 14 wks	53	55	55	56	56	56	30	30	41	52	52	52	39.4 (DHS 2018)
Senegal	2004	2016	B, 6, 10, 14 wks	93	93	92	96	92	86	62	76	81	85	86	78	80.3 (DHS 2019)
Sierra Leone	2007	—	6, 10, 14 wks	84	90	93	95	91	92	NA	NA	NA	NA	NA	NA	83.4 (DHS 2019)
Togo	2008	—	6, 10, 14 wks	82	83	81	84	82	83	NA	NA	NA	NA	NA	NA	80.0 (MICS 2017)
**African Region**	—	—	—	73	74	74	75	73	71	10	10	12	15	16	17	—

**Abbreviations:** B = birth; DHS = demographic health survey; ENAFEME = Enquête Nationale sur la Fécondité et la Mortalité des Enfants de Moins de 5 Ans; HepB = hepatitis B vaccine; HepB-BD = birth dose of monovalent hepatitis B vaccine; HepB3 = third dose of hepatitis B–containing vaccine; IDSR = integrated disease surveillance and response; MICS = multiple indicator cluster survey; MoH = Ministry of Health; NR = not reported; NS = national survey; R = restricted HepB-BD; SHHS = South Sudan Household Health Survey.

* WHO-UNICEF Estimates of National Immunization Coverage. https://immunizationdata.who.int/pages/coverage/HEPB.html

^†^ Timely receipt of HepB-BD is defined as administration of 1 dose of HepB within 24 hours of birth.

^§^ During 2010 to 2018: HepB-BD was selectively given to newborns of mothers who have received a positive HBV? surface antigen test result; in 2019, the country switched to universal HepB-BD vaccination of all newborns.

^¶^ Restricted HepB-BD given only to children born to mothers with hepatitis B.

** Preliminary data.

### HBsAg Seroprevalence Surveys

Because most chronic HBV infections (particularly those among young children) are asymptomatic, the impact of hepatitis B vaccination is usually measured by HBsAg seroprevalence among children born after the introduction of HepB, usually those aged ≤5 years*** (*3*,*6*). During 2016–2021, HBsAg seroprevalence surveys among children were conducted at national or regional levels in eight (17%) countries. Among children of various age ranges surveyed in Ethiopia, Mauritania, Rwanda, Sierra Leone, Uganda, and Zambia, HBsAg seroprevalence was ≤2%. Prevalence among children aged ≤5 years measured in the Democratic Republic of the Congo, Ethiopia, Mauritania, Nigeria, and Sierra Leone ranged from 0.7% (Mauritania) to 4.5% (Nigeria) (Table 2). No country achieved ≤0.1% HBsAg seroprevalence among children. Modeling studies estimated a HBsAg seroprevalence of 2.5% (95% CI = 1.7–4.0) among children aged ≤5 years in AFR, accounting for more than two thirds (4.3 million, approximately 69%) of all infected children worldwide (*1*).

**TABLE 2:** Hepatitis B virus surface antigen seroprevalence based on population-based serosurveys among children and women of reproductive age or pregnant women during antenatal screening in selected countries — World Health Organization African Region, 2016–2021

Survey group, Country	Year of most recent data (source)	Geographic area	Age group	No. of persons tested	HBsAg prevalence, % (95% CI)
**Children born after HepB introduction**					
Democratic Republic of the Congo*	2013–2014 (DHS)	Nationwide	0−5 yrs	277	2.20 (0.3−4.1)
Ethiopia^†^	2017–2018 (PHIA)	Nationwide (Urban)	0−14 yrs^§^	4,729	1.48 (NR)
			5−9 yrs	539	3.34 (NR)
			10−14 yrs	655	3.05 (NR)
Mauritania^¶^	2019–2021 (DHS)	Nationwide	1−4 yrs	2,642	0.70 (NR)
			5−9 yrs	3,447	0.40 (NR)
			10−14 yrs	2,939	2.40 (NR)
Nigeria**	2018 (NAIIS)	Nationwide	2−4 yrs	2,968	4.50 (3.6−5.6)
			5−9 yrs	3,620	6.60 (5.5−7.9)
			2−9 yrs	6,588	5.80 (5.0−6.6)
Rwanda^††^	2018–2019 (PHIA)	Nationwide	10−14 yrs	869	0.00 (NR)
Sierra Leone^§§^	2018 (Household-based survey)	3 of 5 provinces	4−30 mos	1,889	1.30 (0.8−2.0)
			5−9 yrs	2,025	1.60 (1.1−2.3)
Uganda^¶¶^	2016–2017 (PHIA)	Nationwide	0−14 yrs	10,345	0.60 (NR)
Zambia***	2016 (PHIA)	Nationwide	0−14 yrs^†††^	8,015	1.30 (NR)
Women of reproductive age					
Burkina Faso^§§§^	2010–2011 (DHS)	Nationwide	15−49 yrs	8,056	7.80 (7.1−8.6)
Cameroon^¶¶¶^	2017–2018 (PHIA)	Nationwide	15−49 yrs	1,058	6.00 (NR)
Democratic Republic of the Congo*	2013–2014 (DHS)	Nationwide	15−59 yrs	368	3.80 (NR)
Kenya****	2018–2019 (PHIA)	Nationwide	15−49 yrs	1,652	2.70 (NR)
Mauritania^¶^	2019–2021 (DHS)	Nationwide	15−49 yrs	4,420	6.40 (NR)
Nigeria**	2018 (NAIIS)	Nationwide	15−49 yrs	8,682	6.10 (5.1−7.0)
Rwanda^††^	2018–2019 (PHIA)	Nationwide	15−49 yrs	1,813	1.20 (NR)
Sierra Leone^§§^	2018 (Household based survey)	3 of 5 provinces	15−49 yrs	1,776	9.80 (8.1−11.7)
Tanzania^††††^	2016–2017 (PHIA)	Nationwide	15−49 yrs	615	3.70 (NR)
Uganda^¶¶^	2016–2017 (PHIA)	Nationwide	15−49 yrs	1,4716	3.10 (NR)
Zambia***	2016 (PHIA)	Nationwide	15−59 yrs	1,0973	4.10 (NR)
Antenatal screening of pregnant women					
Nigeria^§§§§^	2019 (ANC screening in HIV facilities)	Nationwide (34 of 36 states)	NA	200,473	3.94 (NR)

**Abbreviations:** ANC = antenatal care; DHS = demographic and health survey; HBsAg = hepatitis B virus surface antigen; HepB = hepatitis B vaccine; HIV = human immunodeficiency

virus; NA = not applicable; NAIIS = Nigeria HIV/AIDS Indicator and Impact Survey; NR = not reported; PHIA = population-based HIV impact assessment survey.

* https://www.ncbi.nlm.nih.gov/pmc/articles/PMC6609197/pdf/tpmd180883.pdf

^†^ https://onlinelibrary.wiley.com/doi/full/10.1111/hiv.13457

^§^ Includes children and adolescents aged 11–13 years born before HepB introduction.

^¶^ https://dhsprogram.com/pubs/pdf/FR373/FR373.pdf

** https://global-hepatitis.com/wp-content/uploads/2023/04/GHS2023-Abstract-Book-ONLINE_4.pdf?utm_source=mobile+app&utm_medium=link&utm_campaign=abstract-book (abstract no. 047)

^††^ https://phia.icap.columbia.edu/wp-content/uploads/2020/11/RPHIA-Final-Report_Web.pdf

^§§^ https://www.sciencedirect.com/science/article/pii/S0264410X22003607

^¶¶^ https://phia.icap.columbia.edu/wp-content/uploads/2020/02/UPHIA_Final_Report_Revise_07.11.2019_Final_for-web.pdf

*** https://phia.icap.columbia.edu/wp-content/uploads/2019/03/ZAMPHIA-Final-Report__2.26.19.pdf

^†††^ Includes children and adolescents aged 11–14 years born before the introduction of HepB.

^§§§^ https://www.ncbi.nlm.nih.gov/pmc/articles/PMC6239015/

^¶¶¶^ https://phia.icap.columbia.edu/wp-content/uploads/2021/09/53059-CAMPHIA-Report_EN_WEB_August1.pdf

**** https://phia.icap.columbia.edu/kenya-final-report-2018/

^††††^ https://phia.icap.columbia.edu/wp-content/uploads/2020/02/FINAL_THIS-2016-2017_Final-Report__06.21.19_for-web_TS.pdf

^§§§§^ https://pubmed.ncbi.nlm.nih.gov/34387113/

HBsAg seroprevalence among women of reproductive age or pregnant women provides an estimate of the risk for MTCT of HBV. Data from population-based HBsAg surveys among women of reproductive age or from screening of pregnant women available from 11 countries showed HBsAg seroprevalences ranging from 1.2% (Rwanda) to 9.8% (Sierra Leone) (Table 2).

### Elimination of Mother-to-Child Transmission of HBV

By December 2021, although 21 (45%) AFR countries had developed a plan for EMTCT of HIV, syphilis, and HBV, only six countries^†††^ reported having implemented the EMTCT guidelines for routine HBsAg testing of pregnant women, provision of antiviral medications to eligible (HBsAg-seropositive) women,^§§§^ and administration of HepB-BD to newborns. As of December 2021, ≥90% of pregnant women in 29 (62%) AFR countries had at least one antenatal care visit (Table 3). Data from the most recent nationwide surveys showed that in 37 (79%) countries, approximately one half of women gave birth in health care facilities, and in 23 (49%) countries, ≥80% of women delivered in a health facility (Table 1). To acknowledge progress toward EMTCT of HBV in countries with high endemicity, WHO developed a certification mechanism for the path to elimination of MTCT of HBV, using three tiers (bronze, silver, and gold) indicating increasing levels of progress^¶¶¶^ (*6*). Based on HepB immunization interventions in 2021, Botswana might be eligible for the bronze tier, three countries (Namibia, Sao Tome and Principe, and Senegal) might be eligible for the silver tier, and two countries (Algeria and Cabo Verde) might be eligible for the gold tier certification (Table 1) (Table 3).

**TABLE 3:** Policies and interventions to prevent mother-to-child transmission of hepatitis B virus and tier eligibility* for the path to elimination of mother-to-child transmission of hepatitis B virus — World Health Organization African Region, 2021

Policies and interventions	No. (%) of countries with policy or intervention present or not present	
	Present	Not present
National strategic plan for viral hepatitis^†^	21 (45)	26 (55)
National plan for triple elimination of HIV, syphilis, and hepatitis B^§^	21 (45)	26 (55)
National guidelines for antenatal HBsAg testing and maternal treatment^†^^,^^¶^	17 (36)	30 (64)
ANC1 coverage ≥90%**^,††^	29 (62)	16 (34)
HepB-BD coverage ≥90%^§§^	2 (4)	45 (96)
HepB-BD coverage ≥50%^§§^	6 (13)	41 (87)
HepB3 coverage ≥90%^§§^	16 (34)	31 (66)
Eligibility for bronze tier for path to elimination of MTCT of HBV *^,§§^	1 (2)	—
Eligibility for silver tier for path to elimination of MTCT of HBV *^,§§^	3 (6)	—
Eligibility for gold tier for path to elimination of MTCT of HBV *^,§§^	2 (4)	—

**Abbreviations:** ANC1 = at least 1 antenatal care visit; HBsAg = hepatitis B surface antigen; HBV = hepatitis B virus; HepB-BD = birth dose of monovalent hepatitis B vaccine; HepB3 = three doses of a hepatitis B containing vaccine; MTCT = mother-to-child transmission; WHO = World Health Organization.

* Eligibility for tier certification on the path to elimination of MTCT of HBV is based on immunization interventions. Bronze tier: 1) ≥90% coverage of HepB3 infant vaccination, and 2) implementation of universal timely HepB-BD policy. Silver tier: ≥90% coverage of HepB3 infant vaccination, 2) ≥50% coverage of universal timely HepB-BD, and 3) availability of antenatal HBsAg testing in the public sector. Gold tier: 1) ≥90% coverage of HepB3 infant vaccination, 2) ≥90% coverage of universal timely HepB-BD, and 3) >30% coverage of antenatal HBsAg testing. Indicators for each tier should be achieved for ≥2 years. https://www.who.int/publications/i/item/9789240039360

^†^ https://www.afro.who.int/publications/viral-hepatitis-scorecard-2021-african-region

^§^ All 21 priority countries reported by WHO regional office: Angola, Botswana, Burundi, Cameroun, Chad, Côte d’Ivoire, Democratic Republic of the Congo, Eswatini, Ethiopia, Ghana, Kenya, Lesotho, Malawi, Mozambique, Namibia, Nigeria, Uganda, South Africa, Tanzania, Zambia, and Zimbabwe.

^¶^ Included in national testing and treatment guidelines.

** https://data.unicef.org/resources/dataset/maternal-newborn-health/

^††^ Data are not available for two (4%) countries (Mauritius and Seychelles).

^§§^ WHO-UNICEF estimates. https://immunizationdata.who.int/pages/coverage/HEPB.html

## Discussion

All 47 AFR countries have had HepB in their infant immunization schedule since 2014, and 16 (34%) have achieved ≥90% HepB3 coverage for ≥2 years, including four countries that documented <2% HBsAg seroprevalence in children, consistent with hepatitis B control. The COVID-19 pandemic led to disruptions in immunization services,**** resulting in fewer AFR countries attaining ≥90% HepB3 coverage, declining from a peak of 20 (43%) in 2018 to 16 (34%) in 2021. Strategies to recover and strengthen immunization programs such as catch-up vaccination campaigns, could help ensure that all eligible children who missed HepB vaccination receive the recommended doses (*7*).

Fewer than one third (30%, 14) of countries had introduced HepB-BD by 2021, and just two countries achieved ≥90% HepB-BD coverage. Scaling up HepB-BD introduction and coverage is critical to eliminating MTCT of HBV and preventing subsequent liver disease and associated mortality. During 2016–2021, four countries in AFR introduced HepB-BD which, in addition to increasing HepB-BD coverage in two of these countries (Nigeria and Senegal), resulted in an increase in regional HepB-BD coverage from 10% to 17%. However, in 2021, almost 33 million newborns in AFR did not receive timely HepB-BD. (Table 1) Based on modeled estimates, maintaining current HepB3 coverage and increasing HepB-BD coverage to ≥90% in all countries in the region could avert 554,318 HBV-related deaths among 2020–2030 birth cohorts (*8*). Among the 33 countries that did not have HepB-BD as part of their routine immunization schedules in 2021, two (Burkina Faso and Uganda) introduced it in 2022. Among the remaining 31 countries,^††††^ 13^§§§§^ plan to introduce HepB-BD by 2025.^¶¶¶¶ ^ However, achieving the regional target of 35 countries by 2025 (*5*) would require six to seven countries to introduce HepB-BD each year. Following introduction, delivery in health facilities by skilled workers was shown to be significantly correlated with timely HepB-BD administration (*9*). Promoting and enabling delivery in health facilities, training health care workers, and integrating HepB-BD vaccination into newborn care, are essential to increasing timely HepB-BD coverage in AFR.

In addition to providing timely HepB-BD and HepB3, the identification of pregnant women with HBV infection and provision of antiviral medications for those who are eligible for treatment would further advance EMTCT of HBV (*9*,*10*). However, as of 2021, only 17 (36%) AFR countries had national policies for antenatal HBsAg testing and treatment, and nationally representative serosurveys in AFR were uncommon. HBsAg seroprevalence surveys would help document progress and guide policy decisions regarding hepatitis B control and elimination in the region.

### Limitations

The findings in this report are subject to at least two limitations. First, HepB-BD coverage data were not consistently reported by five countries,***** which might have resulted in the underestimation of overall HepB-BD regional coverage. Second, assessment of hepatitis B control and EMTCT is challenging in countries that have introduced HepB-BD and achieved high coverage with HepB3, because nationally representative seroprevalence surveys to estimate the prevalence of HBV infection among children are lacking in those countries.

### Implications for Public Health Practice

Establishing a regional verification mechanism for hepatitis B control and EMTCT of HBV could elevate the profile of elimination initiatives in AFR. Scaling up the introduction of HepB-BD and strategies to increase timely HepB-BD and HepB3 coverage would accelerate the reduction of preventable hepatitis B–associated morbidity and mortality and progress toward 2030 hepatitis B elimination goals.

## Footnotes

* The African Region, one of the six WHO regions, with a population of approximately 1.2 billion persons, includes 47 countries. https://www.afro.who.int/countries

† Depending on the country’s immunization schedule.

§ Administration of a dose within 24 hours of birth.

¶ HBsAg seropositivity is an indicator of chronic HBV infection.

** Administrative vaccination coverage data are derived from the country’s immunization registry system. The coverage is calculated by dividing the total number of doses administered by the estimated target population for vaccination.

†† Official vaccination coverage estimates are reported by national authorities based on administrative data, immunization coverage surveys, and reports.

§§ 45 C.F.R. part 46, 21 C.F.R. part 56; 42 U.S.C. Sect. 241(d); 5 U.S.C. Sect. 552a; 44 U.S.C. Sect. 3501 et seq.

¶¶ AFR is organized into three functional subregions: Central subregion (Angola, Burundi, Cameroon, Central African Republic, Chad, Democratic Republic of the Congo, Equatorial Guinea, Gabon, Republic of the Congo, and Sao Tome and Principe); East and South subregion (Botswana, Comoros, Eritrea, Eswatini, Ethiopia, Kenya, Lesotho, Madagascar, Malawi, Mauritius, Mozambique, Namibia, Rwanda, Seychelles, South Africa, South Sudan, Uganda, United Republic of Tanzania, Zambia, and Zimbabwe) and West subregion (Algeria, Benin, Burkina Faso, Cabo Verde, Côte d'Ivoire, The Gambia, Ghana, Guinea, Guinea-Bissau, Liberia, Mali, Mauritania, Niger, Nigeria, Senegal, Sierra Leone, and Togo).

*** HBsAg seroprevalence can be measured among children aged 1 year, 5 years, or 1–5 years, according to existing country surveillance and data collection practices. For regions and countries with a long history of high hepatitis B vaccination coverage and those that already conduct school-based serosurveys, serosurveys might be conducted in children aged >5 years. https://www.who.int/publications/i/item/9789240039360

††† Angola, Cabo Verde, Equatorial Guinea, Mozambique, Namibia, and Sao Tome and Principe.

§§§ Pregnant women who received positive HBsAg test results and had an HBV DNA ≥5.3 log10 IU/mL (≥200,000 IU/mL) or received a positive HBsAg antigen test result are recommended by WHO to receive antiviral prophylaxis to prevent MTCT of HBV. https://apps.who.int/iris/bitstream/handle/10665/333391/9789240002708-eng.pdf

¶¶¶ Bronze tier: 1) ≥90% HepB3 infant vaccination coverage, and 2) implementation of universal timely HepB-BD policy for ≥2 years. Silver tier: 1) ≥90% HepB3 infant vaccination coverage, 2) ≥50% universal timely HepB-BD coverage, and 3) availability of antenatal HBsAg testing in the public sector for ≥2 years. Gold tier: 1) ≥90% HepB3 infant vaccination coverage, 2) ≥90% universal timely HepB-BD coverage, and 3) >30% antenatal HBsAg testing coverage for ≥2 years. https://www.who.int/publications/i/item/9789240039360

****
https://www.who.int/publications/i/item/WHO-2019-nCoV-EHS_continuity-survey-2022.1

†††† Burundi, Cameroon, Central African Republic, Chad, Comoros, Democratic Republic of the Congo, Eritrea, Eswatini, Ethiopia, Gabon, Ghana, Guinea, Guinea-Bissau, Kenya, Lesotho, Liberia, Madagascar, Malawi, Mali, Mozambique, Niger, Republic of the Congo, Rwanda, Seychelles, Sierra Leone, South Africa, South Sudan, Togo, United Republic of Tanzania, Zambia, and Zimbabwe.

§§§§ Burundi, Cameroon, Comoros, Eritrea, Ghana, Lesotho, Madagascar, Niger, Seychelle, Sierra Leone, South Africa, Togo, and Zimbabwe.

¶¶¶¶ Obtained from workshop reports on National Immunization Plan; meetings were held during September–October 2022.

***** Angola, Botswana, Equatorial Guinea, The Gambia, and Mauritania.
